# Metabolic flux analysis in hiPSC-CMs reveals insights into cardiac dysfunction in propionic acidemia

**DOI:** 10.1007/s00018-025-05661-5

**Published:** 2025-04-02

**Authors:** Eva Richard, Hannah Marchuk, Mar Álvarez, Wentao He, Xiaoxin Chen, Lourdes R. Desviat, Guo-Fang Zhang

**Affiliations:** 1 Centro de Biología Molecular Severo Ochoa UAM-CSIC, CIBERER, IdiPaz, IUBM, Universidad Autónoma de Madrid, Nicolás Cabrera 1, 28049 Madrid, Spain; 2 Duke Molecular Physiology Institute and Sarah W. Stedman Nutrition and Metabolism Center, Duke University Medical Center, Carmichael Building 48-203, 300 North Duke Street, Durham, NC 27701 USA; 3 Centro de Biología Molecular Severo Ochoa UAM-CSIC, Universidad Autónoma de Madrid, 28049 Madrid, Spain; 4 Surgical Research Lab, Department of Surgery, Cooper University Hospital, Cooper Medical School of Rowan University, Camden, NJ 08103 USA; 5 Coriell Institute for Medical Research, Camden, NJ 08103 USA; 6 MD Anderson Cancer Center at Cooper, Camden, NJ 08103 USA; 7 Department of Medicine, Division of Endocrinology, Metabolism and Nutrition, Duke University Medical Center, Durham, NC 27701 USA

**Keywords:** Propionic acidemia, Human induced pluripotent stem cell-derived cardiomyocytes, Metabolic flux, Glucose metabolism, Fatty acid metabolism, Cardiac diseases

## Abstract

Propionic acidemia is an inborn error of metabolism caused by mutations in either the *PCCA* or *PCCB* genes. Patients with propionic acidemia experience a range of complications, including life-threatening cardiac dysfunctions. However, the pathological mechanisms underlying propionic acidemia-associated cardiac diseases remain largely unknown. To gain insights into the metabolic alterations in propionic acidemia, we studied human induced pluripotent stem cell-derived cardiomyocytes generated from a patient with propionic acidemia with two pathogenic *PCCA* mutations (*p.Cys616_Val633del* and p.Gly477Glufs9*) and from a healthy individual. Using stable isotope-based metabolic flux analysis, we confirmed that the *PCCA* mutations lead to impaired propionyl-CoA carboxylase activity in human induced pluripotent stem cell-derived cardiomyocytes. In addition to being converted to propionylcarnitine, the accumulated propionyl-CoA can also be hydrolyzed to propionate and exported out of the cell, serving as a secondary “pressure valve” to regulate cellular propionyl-CoA levels. Interestingly, the deficiency of propionyl-CoA carboxylase was found to shift fuel metabolism from fatty acid oxidation to increased glucose metabolism human in induced pluripotent stem cell-derived cardiomyocytes from patients with propionic acidemia. This metabolic switch is less energy-efficient and may contribute to the development of chronic cardiac dysfunction in patients with propionic acidemia.

**Supplementary Information:**

The online version contains supplementary material available at 10.1007/s00018-025-05661-5.

## Introduction

Propionic acidemia (PA) is an inborn error of metabolism inherited in an autosomal recessive manner [18, 38, 39, 64]. Mutations in either the *PCCA* or *PCCB* gene result in the malfunction of propionyl-CoA carboxylase (PCC), a critical mitochondrial enzyme. The global incidence of PA varies, ranging from 1 in 50,000 to 1 in 100,000 live births. The adoption of tandem mass spectrometry for neonatal screening has significantly increased the detection rate of PA cases [52].

PCC is a crucial mitochondrial enzyme, though it remains relatively understudied [60]. Whole-body PCC knockout in mouse models has been shown to be neonatal lethal [10, 21, 25, 42]. Patients with PA, often present symptoms in early infancy such as poor feeding, vomiting, and low muscle tone [2, 33]. As the disease progresses, patients may develop a variety of complications, including life-threatening cardiac dysfunctions [31, 32, 59]. Understanding the pathological mechanisms underlying PA-associated cardiac diseases is an urgent and critical need.

PCC catalyzes the carboxylation of propionyl-CoA to methylmalonyl-CoA, which is subsequently converted to succinyl-CoA and enters the tricarboxylic acid (TCA) cycle as part of an anaplerotic process [8, 28, 29, 41, 44]. Malfunctioning PCC disrupts propionyl-CoA metabolism, leading to the accumulation of propionyl-CoA and its metabolites, including methylcitrate, 3-hydroxypropionate, and maleic acid [55, 58]. These accumulated metabolites have been reported to inhibit the TCA cycle and mitochondrial energy production in in vitro enzyme assays using high concentrations of these metabolites [6, 12, 48, 49, 58]. Additionally, oxidative stress, disruption of potassium channels, and altered miRNA expression have been implicated in the cardiac dysfunction observed in patients with PA [7, 11, 17, 19, 20, 47, 50, 52].

Propionyl-CoA, an intracellular metabolite, cannot cross the plasma membrane [56]. Instead, accumulated propionyl-CoA is converted to propionylcarnitine, which can be released into the blood and urine [9, 14]. L-carnitine has been shown to effectively treat PA and is recommended as a supplement for patients [15, 45, 46]. The conversion of propionyl-CoA to propionylcarnitine is considered a critical metabolic pathway for the disposal of accumulated propionyl-CoA, with elevated propionylcarnitine serving as a biomarker for PA. However, PA is also associated with elevated levels of circulating propionate, which is partly due to reduced hepatic disposal [57]. The exact source of circulating propionate remains unclear. While it is known to originate from the microbiome, it is uncertain whether it might also arise from intracellular propionyl-CoA within the host. This could represent a secondary regulatory mechanism for managing intracellular propionyl-CoA, in addition to its conversion to propionylcarnitine.

Previously, we reported that supraphysiological levels of propionate dramatically increase propionyl-CoA and deplete free Coenzyme A (CoA) in perfused rat hearts [55]. This CoA depletion causes a metabolic shift from the high energy-efficient fatty acid oxidation to low energy-efficient glucose metabolism [55]. However, in the *Pcca*^−/−^(A138T) mouse model of PA, CoA or carnitine depletion in the heart occurs only upon the acute administration of high doses of propionate (500 mg/kg) [24]. This highlights the need for a more representative PA model to investigate the pathological mechanisms underlying cardiac diseases associated with PA.

Animal experiments often face limitations in their applicability to humans. In this study, we utilized human induced pluripotent stem cell-derived cardiomyocytes (hiPSC-CMs) from a control individual and a PA patient to investigate metabolic alterations and better understand the pathological mechanisms of PA. Using stable isotope-based metabolic flux analysis, we confirmed the metabolic phenotype of PCC deficiency in PA patient-derived hiPSC-CMs. Our experiments demonstrated that accumulated propionyl-CoA can be hydrolyzed to propionate, which is then exported from the cell as an additional “pressure valve.” Furthermore, PA hiPSC-CMs exhibited a metabolic switch from energy-efficient fatty acid oxidation to energy-inefficient glucose metabolism. This shift in fuel utilization may be a key pathological factor contributing to the cardiac dysfunction observed in PA patients.

## Methods

### Maintenance of hiPSC lines

The hiPSC lines utilized in this study included: (i) a PCCA-deficient hiPSC line (PCCA23-FiPS4F6 or UAMi001-A), created by reprogramming fibroblasts from a patient with *PCCA* gene mutations (c.1899 + 4_1899 + 7delAGTA; p.(Cys616_Val633del) and c.1430-?_1643 + ?del; p.(Gly477Glufs*9)) via Sendai virus; and (ii) a normal control hiPSC line (FIPS Ctrl2-SV4F-1) acquired from the National Bank of Cell Lines at the Carlos III Health Institute (ISCIII, Madrid, Spain).

These human iPSC lines were cultured on 60 mm dishes coated with Matrigel (hESC-qualified matrix, Corning, New York, NY, USA) and maintained in mTESR™ Plus medium (StemCell™ Technologies, Vancouver, BC, Canada), with media changes every other day. The hiPSCs were passaged every four days using ReleSR™ (StemCell™ Technologies) and 10 µM Rock inhibitor (StemCell™ Technologies) at a splitting ratio of 1:3–1:5.

### Differentiation of hiPSCs into cardiomyocytes

hiPSCs cultured in mTESR™ Plus medium were dissociated into single cells using StemPro Accutase (Gibco, Waltham, MA, USA). A total of 1 × 10^6^ cells in 1.5 ml of mTESR™ Plus medium enriched with 10 µM Rock inhibitor were plated on Matrigel-coated 12-well plates. The differentiation into cardiomyocytes was conducted using the STEMdiff™ Cardiomyocyte Differentiation and Maintenance Kits (StemCell™ Technologies) following the guidelines provided by the manufacturer. The characterization of cardiomyocytes was achieved by analyzing the expression of various cardiac-specific markers, including cardiac troponin T (cTnT), α-smooth muscle actin, GATA4, and α-actinin 2 through immunocytochemistry as previously described [4, 5]. The efficiency of generating cTnT-positive cardiomyocytes in both cell lines, as assessed by flow cytometry, was similar (≈ 95%) [4]. In addition, in another study currently under review, we have purified the iPSCs-derived cardiomyocytes for electrophysiological studies. Here, we observed a relatively homogeneous population of ventricular-like cardiomyocytes through action potential recordings. This observation thereby validates our iPSC-CMs differentiation method.

### Experimental conditions and metabolic treatments of hiPSC-derived cardiomyocytes

Control and PA hiPSC-derived cardiomyocytes were subjected to four experimental conditions to assess the impact of various metabolites: Experiment #1: hiPSC-CMs were cultured in RPMI/B27 medium incubated for four days at 37 ℃ in a 5% CO_2_ atmosphere. Experiment #2 (Tracing experiment): hiPSC-CMs were cultured in RPMI/B27 medium with 1 mM [^13^C_3_]propionate (Sigma-Aldrich). Cells were incubated for two days at 37 ℃ in a 5% CO_2_ atmosphere. Experiment #3 (Tracing experiment): hiPSC-CMs were cultured in RPMI/B27 medium without glucose and supplemented with 11 mM [^13^C_6_]glucose (Sigma-Aldrich) and incubated for two days at 37 ℃ in a 5% CO_2_ atmosphere. Experiment #4 (Tracing experiment): hiPSC-CMs were cultured in RPMI/B27 with 0.4 mM [^13^C_16_]palmitate conjugated with BSA (Sigma-Aldrich) medium and incubated for two days at 37 ℃ in a 5% CO_2_ atmosphere. After the incubation period, both the medium and the cell pellets were collected and frozen at − 80 ℃ for subsequent analysis.

### Short-chain fatty acids analysis by LC–MS/MS

An LC–MS/MS method was adapted to analyze short-chain fatty acids including propionate in media [57]. A 30-µl media sample was mixed with 30 µl internal standard (200 µM [2,2,2-^2^H_3_−1,2-^13^C_2_]acetate (D5 acetate), 20 µM [2,2,3,3,3-^2^H_5_]propionate (D5 propionate), 20 µM [2,2,3,3,4,4,4-^2^H_7_]butyrate (D7 butyrate), 20 µM [2,2,3,3,4,4,5,5,5-^2^H_9_]pentanoate (D9 pentanoate), and 20 µM [2,2,3,3,4,4,5,5,6,6,-^2^H_11_]hexanoate (D11 hexanoate)). Acetonitrile (1 ml) was added to precipitate protein. The supernatant was transferred to a new Eppendorf vial and completely dried by nitrogen gas after samples were vortexed and centrifugated at 10,000×g for 20 min. The dried residue was resuspended in 50 µl HPLC water and 20 µl 3-Nitrophenylhydrazine hydrochloride (EDC, 120 mM) and 20 µl (N-(3-Dimethylaminopropyl)-N′-ethylcarbodiimide (3-NPH, 200 mM) for derivatization at 40 ℃ for 30 min. The reaction mixture was centrifuged for 10 min at 10,000×g and the supernatant was transferred to an LC–MS/MS vial for analysis. LC–MS/MS was run with a Sciex QTRAP 6500^+^ MS connected with a Sciex AD UHPLC. An Agilent C18 column (Pursuit XRs C18 150 × 2.0 mm, 5 µm) was employed for separation at room temperature with a flow rate of 0.4 ml/min. A gradient method was conducted with two mobile phases. Mobile phase A was 98% H_2_O and 2% acetonitrile containing 0.1% formic acid. Mobile phase B was 98% acetonitrile and 2% H_2_O containing 0.1% formic acid. The gradient started with 2% B for the first 0.5 min and increased to 90% at 8 min. B was maintained at 90% for 4.5 min and returned to its initial condition within 0.5 min. Finally, the column was re-equilibrated for 9 min with the initial condition before the next injection. The injection volume was 3 µl. MRM in negative mode was used for short-chain fatty acids assay. The MS/MS parameters were set at the following: curtain gas: 35 psi, source temperature: 600 ℃, Gas 1: 55 psi, Gas 2: 55 psi, CAD: 10, Ion spray voltage: − 4500 V, CE: -18 V, EP: − 10 V, and CXP: − 14 V.

### Cell pellets metabolic profiling by GC–MS

A previously established GC–MS method was adopted to measure the isotope labeling of organic acids and amino acids in the cultured pellets [22–24, 55, 57, 62]. Briefly, approximately 1 million cells were spiked with 2 nmol of norvaline and 0.2 nmol [^2^H_9_]L-carnitine or mixed stable isotope labeled metabolites as internal standards and then subjected to sonication extraction with 1 ml methanol for 3 min. The samples were centrifuged for 20 min. The upper phase, approximately 500 µl in volume, was transferred to a fresh Eppendorf vial and subsequently evaporated using nitrogen gas. The resulting dried residues underwent sequential derivatization with methoxylamine hydrochloride and N-tert-butyldimethylsilyl-N-methyltrifluoroacetamide (TBDMS). Specifically, 40 μl of methoxylamine hydrochloride (2% (w/v) in pyridine) was added to the dried residues, followed by incubation for 90 min at 40 ℃. Subsequently, 20 μl of TBDMS with 1% tert-butylchlorodimethylsilane was added, and the mixture was incubated for an additional 30 min at 80 ℃. The derivatized samples were then centrifuged for 10 min at 12,000×g, and the supernatants were transferred to GC vials for further analysis. For GC/MS analysis, we employed an Agilent 7890B GC system with an Agilent 5977A Mass Spectrometer, following the methodology described in our previous work. Specifically, 1 µl of the derivatized sample was injected into the GC column. The GC temperature gradient began at 80 ℃ for 2 min, increased at a rate of 7 ℃ per minute to 280 ℃, and was maintained at 280 ℃ until the 40-min run time was completed. The ionization was conducted via electron impact (EI) at 70 eV, with Helium flow at 1 mL/min. Temperatures of the source, the MS quadrupole, the interface, and the inlet were maintained at 230 ℃, 150 ℃, 280 ℃, and 250 ℃, respectively. Mass spectra (m/z) in the range of 50–700 were recorded in mass scan mode.

### Acylcarnitines profile by LC–MS/MS

A 100 µl medium sample or ~ 1 million cell pellets were used for acylcarnitine assay with the spiked internal standard (20 µl 0.01 mM D9 carnitine). The detailed LC–MS/MS method for the acylcarnitine profile was described in our previous work [23, 24, 55, 57]. The pellet sample extracts (500 µl) from the previous sample preparation were completely dried using nitrogen gas. The medium samples were deproteinized by adding 500 µl methanol and 500 µl acetonitrile. After centrifugation at 12,000 g for 15 min, the supernatants were completely dried using nitrogen gas. The dried residues were then methylated with a 3 M HCl methanol solution (100 µl) at 50 °C for 25 min. After methylation, the samples were once again dried completely using nitrogen gas and then reconstituted in 20 µl of methanol and 60 µl of water. The derivatized samples were subsequently analyzed using an LC-QTRAP 6500^+^-MS/MS (Sciex, Concord, Ontario). A gradient HPLC method with two mobile phases (mobile phase A was 98% water with 2% acetonitrile and 0.1% formic acid and mobile phase B was 98% acetonitrile with 2% H_2_O and 0.1% formic acid) was adopted to run with an Agilent Pursuit XRs 5 C18 column (150 × 2.0 mm). The gradient started with 0% B within first 2 min and then increased to 80% at 13 min. The column was washed out by 90% B for 4 min and equilibrated with initial condition (2% B) for 5 min before next injection. The flow rate was 0.4 ml/min and the column oven was set at room temperature. The injection volume was 2 µl. The parameters for Sciex QTRAP 6500^+^ mass spectrometry were optimized as following: DP: 33 V, CE: 24 V, EP: 10 V, CXP: 10 V, source temperature: 680 ℃, gas 1: 65, gas 2: 65, curtain gas: 35, CAD: 10, and ion spray voltage: 5500 V. The Q1 of all the methylated acylcarnitines was scanned from m/z 218 to m/z 444 with the same fragment (Q3) at m/z 99. L-carnitine had the ion transition of Q1 (m/z 176) and Q3 (m/z 85 or m/z 117). [^2^H_9_]L-carnitine has the shifted Q1 at m/z 185 with the same Q3 at m/z 85 or m/z 117.

### Medium glucose assay by LC-Q-Exactive^+^-MS

Glucose in the medium was assayed according to our previous method. Briefly, a 10 µl of medium sample was added to a tube prior to folch extraction using the following solvents: 200 µl methanol, 200 µl distilled H_2_O, and 200 µl chloroform. The sample mixture was vortexed and centrifuged for 20 min at 10,000 × g at 4 ℃. The upper phase (~ 350 µl) was dried completely by nitrogen gas at 37 ℃. The dried residue was resuspended in 60 µl distilled water, vortexed, and placed in an autosampler vial for LC–MS analysis.

LC-Q-Exactive^+^-Orbitrap-MS was used for the final quantitation in this work. The Vanquish Binary Pump was used to deliver the mobile phase (98% H_2_O and 2% methanol containing 0.01% formic acid) at a flow rate of 0.3 ml/min in isocratic elution mode. The column was a Microsorb-MV C18 column (100 × 4.6 mm, 3 µm) with a C18 guard column and was kept at 40 °C in the column oven compartment. The autosampler was maintained at 5 ℃, and the injection volume was 1 µl. The total running time is 10 min. The parameters for Q-Exactive^+^-MS equipped with a HESI probe: heat temperature: 425 ℃; sheath gas: 30, auxiliary gas, 13; sweep gas, 3; spray voltage, 3.5 kV for positive mode; the capillary temperature was set at 320 ℃, and S-lens was 45. A full scan range was set at 60–900 (m/z). The resolution was set at 70,000 (at m/z 200). The maximum injection time (max IT) was 200 ms. Automated gain control (AGC) was targeted at 3 × 10^6^ ions.

### Statistics

All cell experiments were conducted using two differentiations with a total of n = 4 biological replicates. Measured mass isotopologues distributions expressed as mol percent were corrected for natural enrichment [16, 53]. M0, M1, M2, …, Mn denote the isotopologues of molecules containing n heavy atoms. Statistical differences were analyzed using Prism software. A Student’s t test was used for comparisons between two groups.

## Results

### Metabolic profile of human induced pluripotent stem cell-derived cardiomyocytes from a control individual and a PCCA-deficient patient

**Fig. 1:**
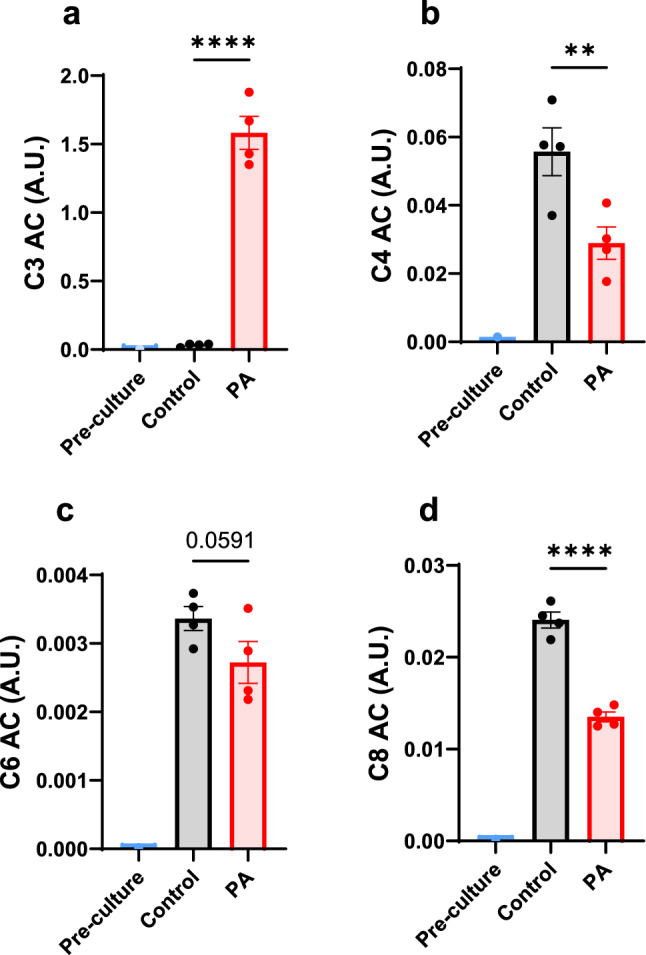
Release of acylcarnitines from hiPSC-CMs into the culture medium. **a–d** Levels of propionylcarnitine (C3 AC), butyrylcarnitine (C4 AC), hexanoylcarnitine (C6 AC), and octanoylcarnitine (C8 AC) in the culture medium after a 4-day incubation with RPMI/B27 medium. Data are presented as mean ± SE, with N = 4 per group. ** and **** indicate p-values < 0.01 and < 0.001, respectively. The corresponding t-values for panels **a**, **b**, **c**, and **d** are 12.9, 3.2, 1.8, and 10.2

Metabolic profiling was performed on control and PCCA-deficient hiPSC-CMs using both cell pellets and cultured media samples. PCC deficiency significantly increased propionylcarnitine (C3 AC) in the cultured media (Fig. 1a) and led to the accumulation of cellular methylcitrate (Supplemental Fig. 1a). Interestingly, medium-chain acylcarnitines were found to be lower in the cultured media from the PCCA-deficient group (Fig. 1b–d). This decrease in acylcarnitines may indicate reduced fatty acid oxidation or a redistribution of acylcarnitines toward increased propionylcarnitine synthesis. In line with this, there was a trend toward a reduction in cellular L-carnitine (p = 0.076, Supplemental Fig. 1b). The accumulation of propionylcarnitine, along with the reduction of free carnitine, could alter fuel metabolism in the cardiomyocytes, which depend on proper fuel metabolism for mechanical contraction.

### PCC deficiency disrupts propionate and propionyl-CoA metabolism in hiPSC-CMs

**Fig. 2:**
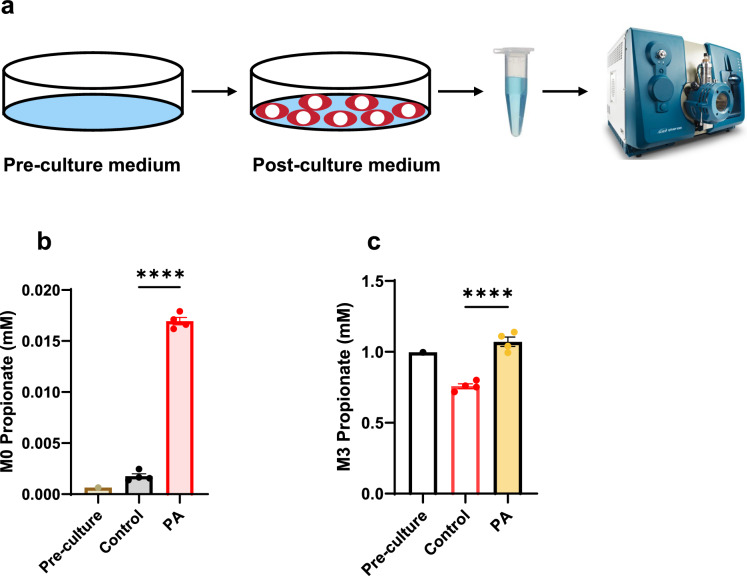
Effect of PCC deficiency on [^13^C_3_]propionate consumption. **a** Schematic representation of the experimental design and procedure. **b** Levels of unlabeled propionate (M0 propionate) in the pre-culture medium and post-culture medium of hiPSC-CMs derived from healthy controls (Control) and PA patients (PA). **c** Residual [^13^C_3_]propionate (M3 propionate) in the culture medium after 2 days of incubation with 1 mM [^13^C_3_]propionate in RPMI/B27 medium, comparing hiPSC-CMs from Control and PA groups. Data are presented as mean ± SE, with N = 4 per group. **** indicates p-values < 0.001. The corresponding t-values for panels **b** and **c** are 34.6 and 8.4

One of the reliable biomarkers of PA is elevated circulating propionate, which can reach millimolar levels in PA patients [26, 57]. However, this biomarker is not often reported due to challenges with analytical methodology. Our previous work suggests that impaired hepatic disposal of propionate leads to increased circulating levels, which may elevate the risk of cardiac disease [57]. In the present study, we aimed to investigate the impact of PCC deficiency on propionate metabolism in cardiomyocytes derived from both control and PCCA-deficient hiPSC-CMs. After culturing the cells for 48 h with 1 mM [^13^C_3_]propionate, both pre-culture and post-culture media were collected for propionate quantification (Fig. 2a). PCCA-deficient hiPSC-CMs released 9.6 times more unlabeled propionate compared to control hiPSC-CMs (Fig. 2b). The released unlabeled propionate is likely generated endogenously, probably from hydrolysis of propionyl-CoA, which is elevated in PA patient-derived hiPSC-CMs. After 48 h of culturing, [^13^C_3_]propionate was consumed by 25% in the control hiPSC-CMs (Fig. 2c). In contrast, [^13^C_3_]propionate levels in the medium remained almost unchanged in PA hiPSC-CMs (Fig. 2c). This strongly suggests that the PCC deficiency not only affects propionyl-CoA metabolism but also disrupts propionate metabolism, consistent with our previous findings in the livers of *Pcca*^−/−^(A138T) mice [57].

**Fig. 3:**
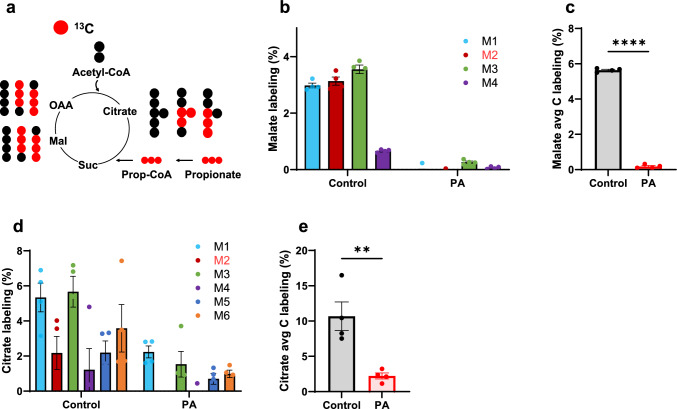
PCC deficiency reduces [^13^C_3_]propionate metabolism. **a** Simplified schematic of the [^13^C_3_]propionate tracing pathway during the first round of TCA cycle entry. **b**, **c** Stable isotopomer labeling and average carbon (C) labeling of malate. **d**, **e** Stable isotopomer labeling and average carbon (C) labeling of citrate. hiPSC-CMs from control individual and PA patient were incubated with 1 mM [^13^C_3_]propionate for 48 h. Data are presented as mean ± SE, with N = 4 per group. ** and **** represent p-values < 0.01 and < 0.001, respectively. The corresponding t-values for panels **c** and **e** are 74.4 and 4.1

[^13^C_3_]Propionate, used as a tracer, allows for tracking both propionate and propionyl-CoA metabolism [24]. TCA cycle intermediates are downstream metabolites of propionyl-CoA, and stable isotope labeling of these intermediates reflects propionyl-CoA metabolism (Fig. 3a). The stable isotopomer labeling and average carbon labeling of malate and citrate are shown in Fig. 3b–e. The low labeling of malate and citrate in PA hiPSC-CMs confirms that the PCCA mutation impairs PCC activity and demonstrates that this disrupts propionyl-CoA anaplerosis into the TCA cycle. The dramatic reduction in labeling of TCA cycle intermediates is further corroborated by other metabolite labeling data (Supplemental Fig. 2).

### PCC deficiency enhances glucose metabolism in hiPSC-CMs

**Fig. 4:**
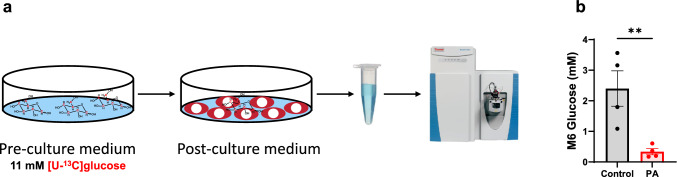
PCC deficiency increases [^13^C_6_]glucose consumption. **a** Schematic representation of the experimental design and procedure for measuring [^13^C_6_]glucose consumption from pre-culture and post-culture media. **b** [^13^C_6_]Glucose concentrations in the culture media after 48 h of incubation with hiPSC-CMs derived from a healthy control individual and a PA patient, using 11 mM [^13^C_6_]glucose. Data are presented as mean ± SE, with N = 4 per group. ** indicates p-values < 0.01. The corresponding t-values for panel **b** is 3.5

Approximately 25% or more of patients with PA develop cardiac diseases, although the pathological mechanisms remain largely unknown [35]. Fuel metabolism is crucial for cardiac function, as it sustains energy production required for mechanical contraction. To better understand the metabolic alterations in PA, we investigated the major fuel metabolism pathways in PCCA-deficient hiPSC-CMs. We replaced unlabeled glucose (11 mM) with 11 mM [^13^C_6_]glucose in the medium to trace glucose metabolism. First, we measured the consumption of [^13^C_6_]glucose by quantifying the levels of [^13^C_6_]glucose in both pre- and post-culture media using LC-Q Exactive^+^-MS (Fig. 4a). After 48 h of incubation, [^13^C_6_]glucose levels were much lower in the medium of PA hiPSC-CMs compared to control hiPSC-CMs (Fig. 4b), indicating that PA hiPSC-CMs metabolize [^13^C_6_]glucose at a higher rate than control cells.

**Fig. 5:**
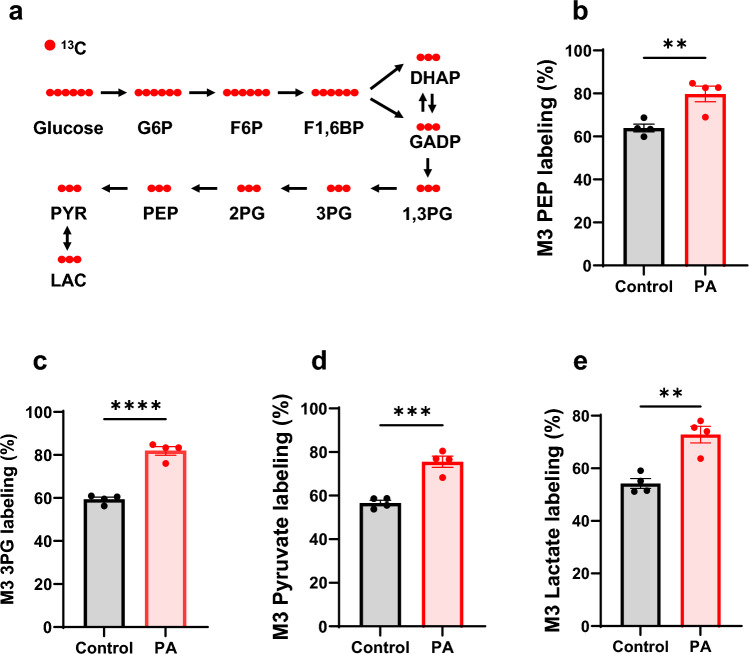
PCC deficiency enhances [^13^C_6_]glucose glycolysis. **a** Schematic representation of [^13^C_6_]glucose metabolism through glycolysis, showing the labeling pathway. G6P, F6P, DHAP, GADP, 1,3PG, 2PG, PYR, and LAC represent glucose-6-phosphate, fructose-6-phosphate, fructose-1,6-bisphosphate, dihydroxyacetone phosphate, glyceraldehyde 3-phosphate, 1,3-bisphosphoglycerate, 2-phosphoglycerate, pyruvate, and lactate, respectively. **b–e** M3 isotopomer labeling of glycolytic intermediates, including phosphoenolpyruvate (PEP), 3-phosphoglycerate (3PG), pyruvate, and lactate, in hiPSC-CMs from the control individual and the PA patient after 48 h of culture with 11 mM [^13^C_6_]glucose. Data are presented as mean ± SE, with N = 4 per group. **, ***, and **** indicate p-values < 0.01, < 0.005, and < 0.001, respectively. The corresponding t-values for panels **b**, **c**, **d** and **e** are 3.9, 10.0, 6.6 and 5.1

Using ^13^C labeled tracer, we traced the downstream metabolites of glucose to assess glucose metabolic rate through stable isotopomer analysis (Fig. 5a). The stable isotope labeling of glycolytic intermediates—such as 3-phosphoglycerate (3PG), phosphoenolpyruvate (PEP), pyruvate, and lactate—was measured (Fig. 5b–e). The stable isotope labeling of these intermediates was significantly higher in PA hiPSC-CMs, reaching 80%, compared to 60% in control hiPSC-CMs. This confirms that glucose metabolism is elevated in PA-derived cardiomyocytes.

**Fig. 6:**
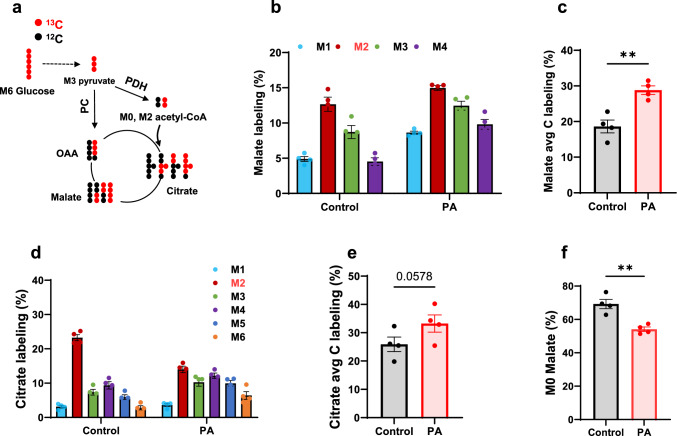
PCC deficiency enhances [^13^C_6_]glucose metabolism through the TCA cycle. **a** Schematic representation of [^13^C_6_]glucose metabolism through the first round of the TCA cycle, showing [^13^C_3_]pyruvate entry via pyruvate dehydrogenase (PDH) and pyruvate carboxylase (PC). Key intermediates include acetyl-CoA, citrate, malate and oxaloacetate (OAA). **b–e** Stable isotopomer labeling and average carbon (C) labeling of malate and citrate derived from [^13^C_6_]glucose in hiPSC-CMs from the control individual and the PA patient after 48 h of culture. **f** Percentage of unlabeled malate in the same samples. Data are presented as mean ± SE, with N = 4 per group. ** indicates p-values < 0.01. The corresponding t-values for panels **c**, **e**, and **f** are 4.7, 1.8, and 4.9

Further downstream in the glucose metabolic pathway, TCA cycle intermediates were labeled by [^13^C_6_]glucose (Fig. 6a). Figure 6b–e show the stable isotopomer labeling and average carbon labeling of malate and citrate in control and PA hiPSC-CMs. Consistent with the increased glucose metabolism in PA hiPSC-CMs, the stable isotope labeling of malate was significantly higher in PA than in control cells. Additionally, the shift to a higher isotopomer of citrate in PA hiPSC-CMs demonstrates increased metabolic flux of glucose into the TCA cycle (Fig. 6d). The lower M2 citrate in PA hiPSC-CMs also supports the finding of reduced unlabeled oxaloacetate/malate (Fig. 6f), as M2 citrate is primarily derived from M0 oxaloacetate and M2 acetyl-CoA (M2 citrate = M0 oxaloacetate × M2 oxaloacetate). This pattern was also observed in other detected TCA cycle intermediates (Supplemental Fig. 3).

Interestingly, M3 malate was the second most abundant isotopomer after M2 (Fig. 6b). The minor amount of M3 malate could be generated from multiple turns of the TCA cycle after M2 acetyl-CoA enters the cycle (Supplement Fig. 4). M2 malate and M3 malate are primarily derived from M2 acetyl-CoA via pyruvate dehydrogenase and M3 pyruvate via pyruvate carboxylase, respectively. The increased glucose metabolism in PA hiPSC-CMs is likely driven by enhanced metabolic flux through both pyruvate dehydrogenase and pyruvate carboxylase.

### Increased secretion of labeled acetate and unlabeled propionate in PA hiPSC-CMs

**Fig. 7:**
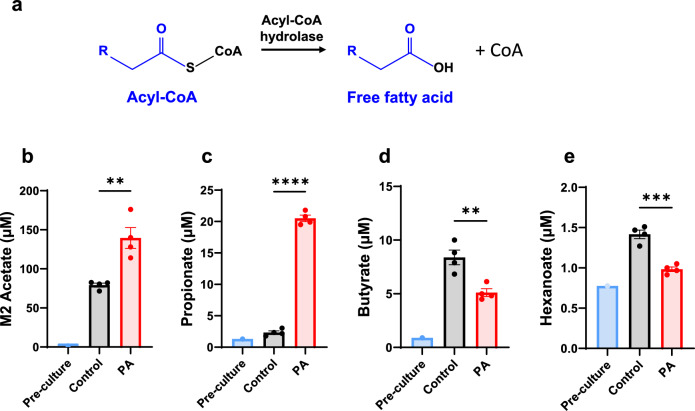
Short-chain fatty acids are hydrolyzed from acyl-CoAs in hiPSC-CMs. **a** Schematic representation of acyl-CoA hydrolase-mediated hydrolysis of acyl-CoAs into free fatty acids. **b** Concentration of M2 acetate in the cultured media from control and PA hiPSC-CMs after 48 h of incubation with [^13^C_6_]glucose. **c–e** Concentrations of unlabeled propionate, butyrate, and hexanoate in the pre- and post-cultured media from control and PA hiPSC-CMs after 48 h of incubation with [^13^C_6_]glucose. Data are presented as mean ± SE, with N = 4 per group. ** and *** denote p-values < 0.01 and < 0.005, respectively. The corresponding t-values for panels **b**, **c**, **d**, and **e** are 4.4, 31.6, 4.3, and 7.3

Acyl-CoAs are intracellular metabolites that cannot cross the plasma membrane [56]. To prevent the accumulation of acyl-CoAs, which can be toxic due to their detergent-like effect, they are converted into their counterparts, acylcarnitines. This metabolic conversion serves as a protective mechanism. In the context of PA, carnitine supplementation can facilitate the conversion of accumulated propionyl-CoA to propionylcarnitine, helping to remove excess propionyl-CoA from cells. However, it remains unclear whether acyl-CoAs could also be hydrolyzed into free fatty acids, offering an additional means for the intracellular metabolites to escape cells and enter the bloodstream (Fig. 7a). In this study, we measured short-chain fatty acids in the cultured media and detected M2 acetate, derived from labeled M2 acetyl-CoA, in the media from [^13^C_6_]glucose (Fig. 7b). This confirmed the activity of acyl-CoA hydrolase, which plays a secondary role in regulating cellular acyl-CoA levels. The increased secretion of labeled acetate (M2 acetate) from [^13^C_6_]glucose in PA hiPSC-CMs also suggests enhanced glucose metabolism in these cells. As anticipated, accumulated propionyl-CoA led to the increased release of propionate into the medium (Fig. 7c) via acyl-CoA hydrolase, consistent with results from the [^13^C₃]propionate experiment (Fig. 2b). In contrast, the release of butyrate and hexanoate decreased, consistent with the reduction of medium-chain acylcarnitines (Figs. 7d, e, 1b–e). This decrease in butyrate, hexanoate, and their corresponding acylcarnitines further supports the shift from fatty acid metabolism to glucose metabolism in PA hiPSC-CMs.

### Reduced fatty acid oxidation in PA hiPSC-CMs

**Fig. 8:**
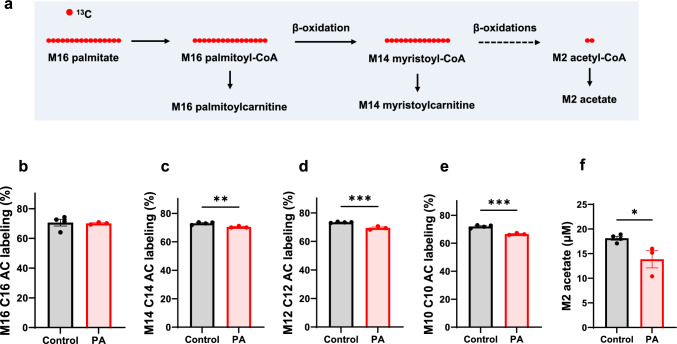
PCC deficiency reduces [^13^C_16_]palmitate metabolism**.** (**a**) Schematic representation of [^13^C_16_]palmitate metabolism. (**b-f**) Stable isotopomer labeling of M16 palmitoylcarnitine (M16 C16 AC), M14 myristoylcarnitine (M14 C14 AC), M12 lauroylcarnitine (M12 C12 AC), M10 caproylcarnitine (M10 C10 AC), and M2 acetate in the cultured media from control and PA hiPSC-CMs after 48 h of incubation with 0.4 mM [^13^C_16_]palmitate. Data are presented as mean ± SE, with N = 4 per group. *, **, and *** indicate p-values < 0.05, < 0.01, and < 0.005, respectively. The corresponding t-values for panels **c**, **d**, **e**, and **f** are 4.3, 6.6, 7.6, and 2.8

The increased lipid droplets have been observed in the heart from patients with PA [34]. A recent study using stable isotope techniques also demonstrated altered lipid metabolism in PA patients [51]. Given the increased glucose metabolism, we sought to investigate fatty acid metabolism in PA hiPSC-CMs using [^13^C_16_]palmitate. After 48 h of culturing, we measured the labeling of metabolites derived from [^13^C_16_]palmitate (Fig. 8a). In the cultured medium samples, acylcarnitine intermediates from M16 palmitate were significantly lower in PA hiPSC-CMs (Fig. 8b–e and Supplemental Fig. 5a–c). Notably, M2 acetate labeling was also significantly lower in the cultured media of PA hiPSC-CMs (Fig. 8f). As observed in the [^13^C_6_]glucose tracing experiment, M2 acetate is derived from the hydrolysis of M2 acetyl-CoA, which is produced during the metabolism of both [^13^C_6_]glucose and [^13^C_16_]palmitate. However, in contrast to the [^13^C_6_]glucose experiment (Fig. 7b), M2 acetate labeling in PA hiPSC-CMs was significantly lower than in control cells when [^13^C_16_]palmitate was used as a tracer (Fig. 8f). Together, these findings suggest that PCC deficiency induces a fuel switch from fatty acid metabolism to increased glucose utilization.

Free carnitine plays a crucial role in transporting long-chain fatty acids into the mitochondria for complete β-oxidation. We observed a reduction, or a trend toward reduced, cellular L-carnitine in all experiments (Supplemental. Figure 1b and Supplemental Fig. 6). The L-carnitine deficiency in PA hiPSC-CMs impedes fatty acid oxidation, which in turn promotes increased glucose metabolism.

## Discussion

PA is a rare metabolic disorder caused by mutations in *PCCA* or *PCCB* genes, resulting in impaired propionyl-CoA metabolism. PA is typically diagnosed early in life, often within days of birth. If left unmanaged, PA can lead to severe complications, including cardiac dysfunction. The underlying mechanisms driving these cardiac complications are not yet fully understood. Chronic metabolic alterations associated with PA are believed to contribute to these complications.

Energy metabolism is crucial for the heart to perform mechanical work. Fatty acids are the heart’s preferred and most efficient fuel for ATP production. In failing hearts, however, fuel metabolism switches from fatty acids to increased glucose and ketone metabolism [13]. This shift may be particularly pronounced in PA patients due to several factors: (1) the depletion of L-carnitine and free CoA, caused by the accumulation of propionylcarnitine and propionyl-CoA, which impairs fatty acid oxidation, (2) the accumulation of lipid droplets in the heart, indicating disrupted fatty acid metabolism [4, 5], and (3) the inhibition of fatty acid oxidation during exercise in PA patients [51]. In this study, we observed that PA hiPSC-CMs exhibit a significant increase in glucose utilization and a decrease in fatty acid oxidation using stable isotope-based metabolic flux analysis. This chronic metabolic switch could impair cardiac energy metabolism and lead to progressive cardiac dysfunction, as the heart preferentially utilizes fatty acids for energy. Interestingly, while resting fatty acid oxidation in PA patients may not differ significantly from healthy individuals, PA patients are unable to fully utilize fatty acids during exercise when the whole-body switches to fatty acid oxidation [51].

The accumulation of propionylcarnitine in PA leads to decreased intracellular L-carnitine levels, particularly in tissues like the heart. One PA patient with fatal cardiomyopathy had low carnitine levels in the heart despite supplementation [40]. Reduced intracellular l-carnitine (Supplemental Fig. 1b and Supplemental Fig. 6) limits the transport of long-chain fatty acids into mitochondria for complete β-oxidation. Chronic reductions in fatty acid oxidation could trigger cardiac disease, as seen in patients with long-chain fatty acid β-oxidation disorders, who frequently develop cardiomyopathy [30].

With [^13^C_6_]glucose as the tracer, we observed increased labeling of both glycolysis and TCA cycle intermediates, indicating enhanced glucose metabolism at both stages of glycolysis and complete TCA cycle oxidation. However, based on the ^13^C labeling data presented here, we cannot determine whether TCA cycle flux is affected by propionyl-CoA or its metabolites in PA hiPSC-CMs, as previously reported [6, 12, 48, 54, 61].

Propionate in plasma and urine is another reliable biomarker for PA. Short-chain fatty acids (SCFAs), typically produced by the microbiome, are usually at low levels in the portal vein of germ-free mice [23, 57]. The liver plays a key role in metabolizing and disposing of propionate, maintaining low levels (~ < 1 µM) [57]. However, it remains unclear whether SCFAs could be produced endogenously, given two key observations: (1) acetate, the most abundant SCFA in the blood, correlates well with acetyl-CoA levels, which are among the highest of all acyl-CoAs, and (2) propionate arises in PA due to propionyl-CoA accumulation. This raises the question of whether acyl-CoA hydrolysis might be another source of SCFAs.

Acyl-CoA hydrolysis, a process that releases free fatty acids that can be transported out of cells, is a potential regulatory mechanism that controls intracellular acyl-CoA levels and prevents their accumulation. Although the role of acyl-CoA hydrolases is understudied, they could play an essential role in regulating CoA for other metabolic events in the cell [1, 3]. In peroxisomes, acyl-CoAs are hydrolyzed into free fatty acids, which are then transported to mitochondria for complete β-oxidation [63]. We confirmed the acyl-CoA hydrolysis activity using stable isotope tracing in this study. Labeled acetate and unlabeled propionate, hydrolyzed from labeled acetyl-CoA and unlabeled propionyl-CoA, occur in mitochondria, where pyruvate oxidation to acetyl-CoA and propionyl-CoA metabolism predominantly take place. Free acetate from pyruvate metabolism could also be directly catalyzed by pyruvate dehydrogenase or 2-ketoglutarate dehydrogenase according to Liu et al.’s work [37]. However, the release of propionate, butyrate and hexanoate confirms the acyl-CoA hydrolase activity. Short-chain fatty acids including propionate released to be out of cells probably can freely cross mitochondrial membranes but needs the organic anion transporter or monocarboxylate transporters in plasma membrane [27, 36, 43].

The physiological implications of propionyl-CoA hydrolysis into propionate in PA warrant further investigation. This hydrolysis could represent a second regulatory mechanism to control intracellular propionyl-CoA levels, serving as a “pressure valve” to mitigate the toxicity of accumulated propionyl-CoA in mitochondria.

This study is the first to utilize cardiomyocytes derived from patients with PA for metabolic discovery, emphasizing its strong clinical relevance. However, the authors acknowledge the following limitations: First, although the propionyl-CoA accumulation-induced metabolic switch is consistent with our previous findings in the perfused rat heart [55], other factors unrelated to the PCCA mutation may contribute to this metabolic shift. This issue could be addressed by using an isogenic cell line; however, the large deletions present in the hiPSC-CMs derived from the PA patient renders the generation of an isogenic line largely unfeasible. Future studies employing other isogenic cell lines or transfecting a wild-type-copy of the *PCCA* gene to analyze the restoration of the metabolic phenotype are warranted to overcome this limitation. Second, this is an in vitro experiment, which does not fully capture the complexity of metabolic regulation present in vivo. The in vitro conditions may not accurately reflect the physiological environment in vivo. These limitations highlight the need for future in vivo studies, which face the following challenges: (1) Metabolic data from the heart in vivo is often confounded by secondary metabolites originating from other organs. (2) Experimental findings using heart tissue from animal models may not directly translate to human patients.

In summary, using stable isotope tracing and hiPSC-CMs derived from a PCCA patient, we provide the direct evidence that PA induces a fuel switch from fatty acids to glucose in iPSCs- derived cardiomyocytes from patients with PA. This shift may underlie the cardiac complications observed in PA. Additionally, we identify a second metabolic regulatory mechanism—acyl-CoA hydrolysis—that helps control propionyl-CoA levels in PA. This pathway may offer a potential therapeutic target for treating PA.

## Supplementary Information

Below is the link to the electronic supplementary material.

Supplementary file1 (PPTX 1027 KB)

## Data Availability

Data will be made available from authors on request.
