# Supplementary material for: Metabolic flux analysis in hiPSC-CMs reveals insights into cardiac dysfunction in propionic acidemia
Source: Cell Mol Life Sci. 2025 Apr 2;82(1):137. doi: 10.1007/s00018-025-05661-5 (PMC11965053; doi:10.1007/s00018-025-05661-5)
Supplement: Supplementary file 1 — Supplementary file1 (PPTX 1027 KB) [file 18_2025_5661_MOESM1_ESM.pptx]

## Slide 1
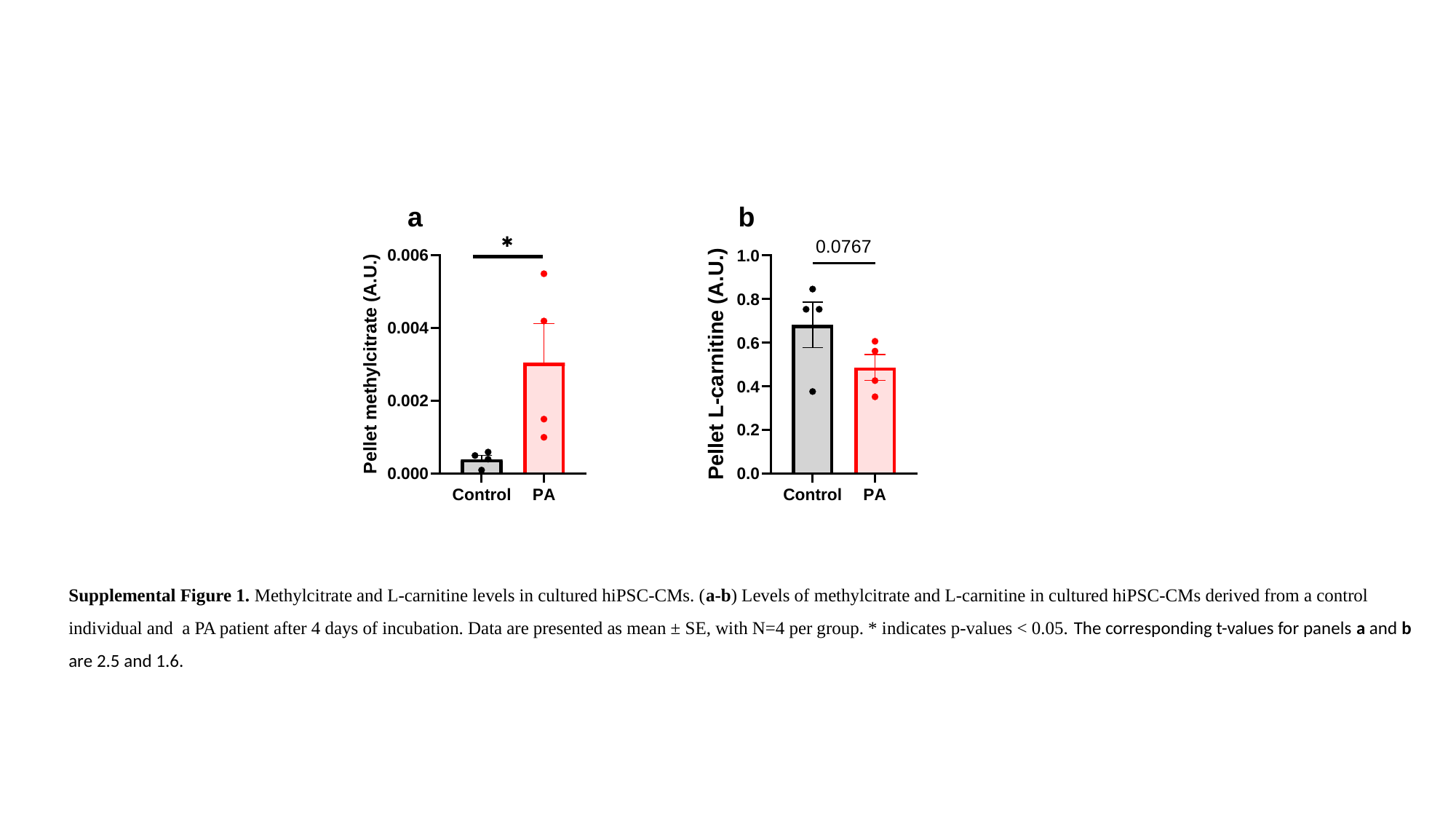

a
b
Supplemental Figure 1. Methylcitrate and L-carnitine levels in cultured hiPSC-CMs. (a-b) Levels of methylcitrate and L-carnitine in cultured hiPSC-CMs derived from a control individual and a PA patient after 4 days of incubation. Data are presented as mean ± SE, with N=4 per group. * indicates p-values < 0.05. The corresponding t-values for panels a and b are 2.5 and 1.6.

## Slide 2
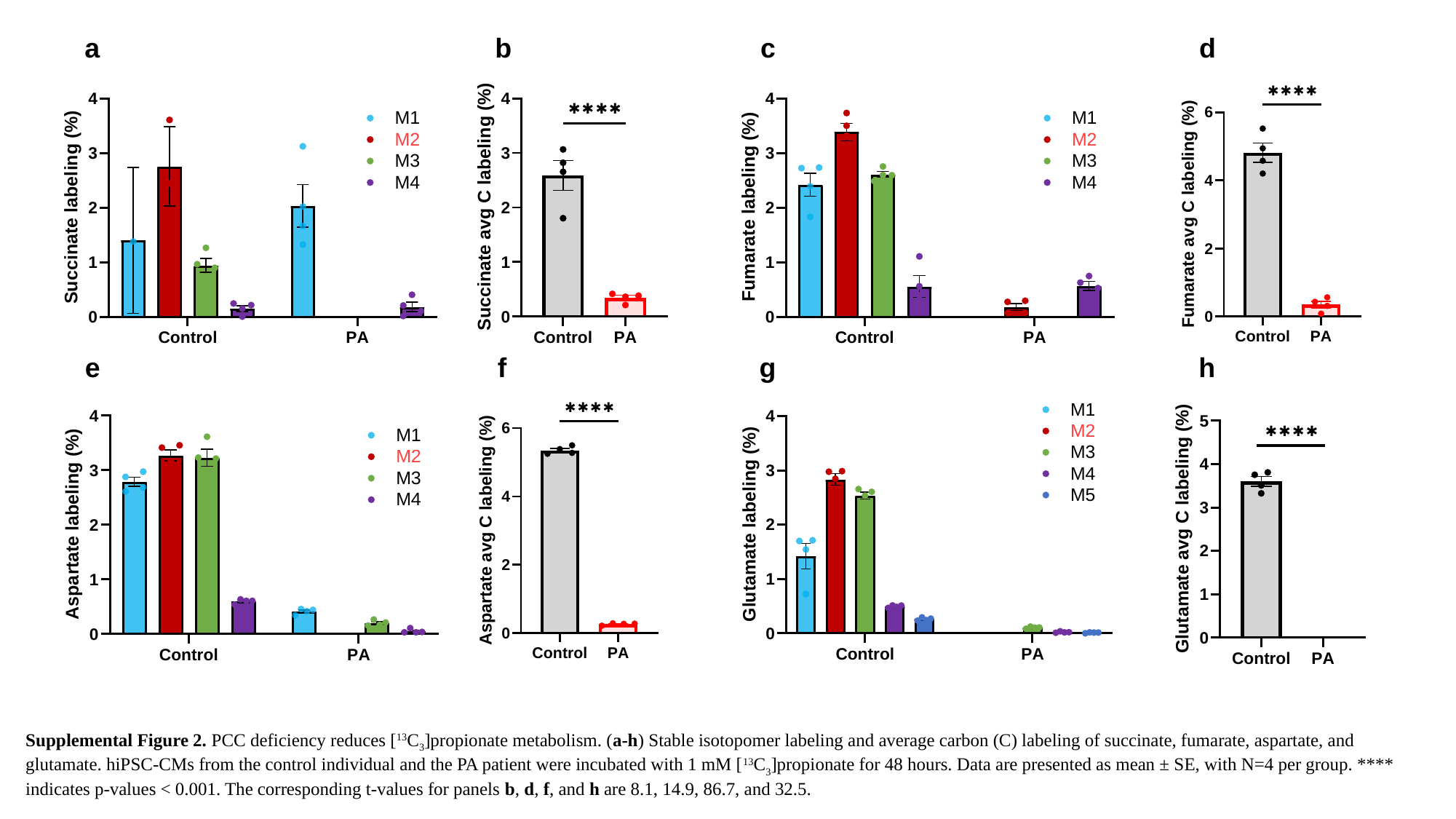

a
b
c
d
e
f
g
h
Supplemental Figure 2. PCC deficiency reduces [13C3]propionate metabolism. (a-h) Stable isotopomer labeling and average carbon (C) labeling of succinate, fumarate, aspartate, and glutamate. hiPSC-CMs from the control individual and the PA patient were incubated with 1 mM [13C3]propionate for 48 hours. Data are presented as mean ± SE, with N=4 per group. **** indicates p-values < 0.001. The corresponding t-values for panels b, d, f, and h are 8.1, 14.9, 86.7, and 32.5.

## Slide 3
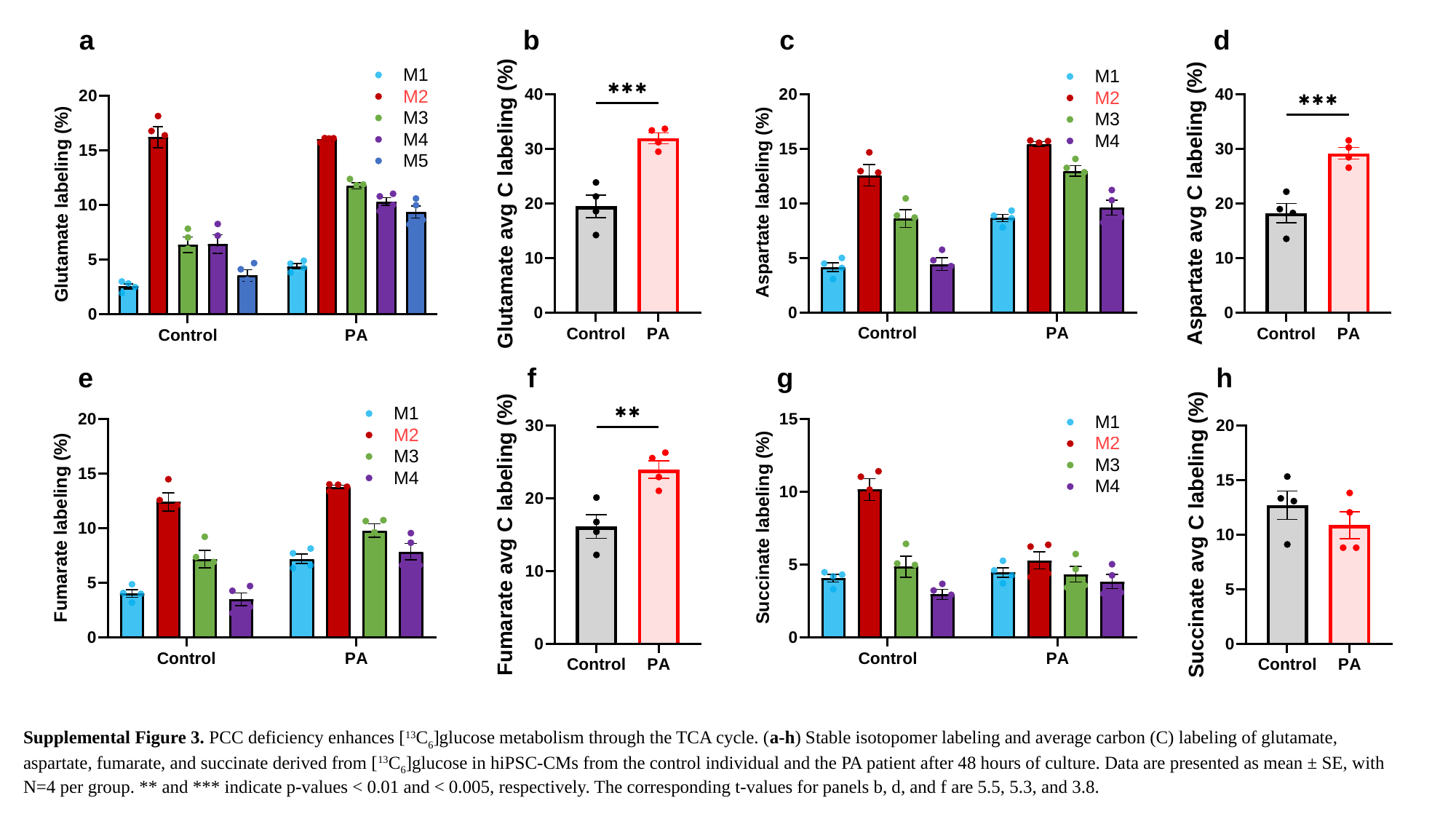

a
b
c
d
e
f
g
h
Supplemental Figure 3. PCC deficiency enhances [13C6]glucose metabolism through the TCA cycle. (a-h) Stable isotopomer labeling and average carbon (C) labeling of glutamate, aspartate, fumarate, and succinate derived from [13C6]glucose in hiPSC-CMs from the control individual and the PA patient after 48 hours of culture. Data are presented as mean ± SE, with N=4 per group. ** and *** indicate p-values < 0.01 and < 0.005, respectively. The corresponding t-values for panels b, d, and f are 5.5, 5.3, and 3.8.

## Slide 4
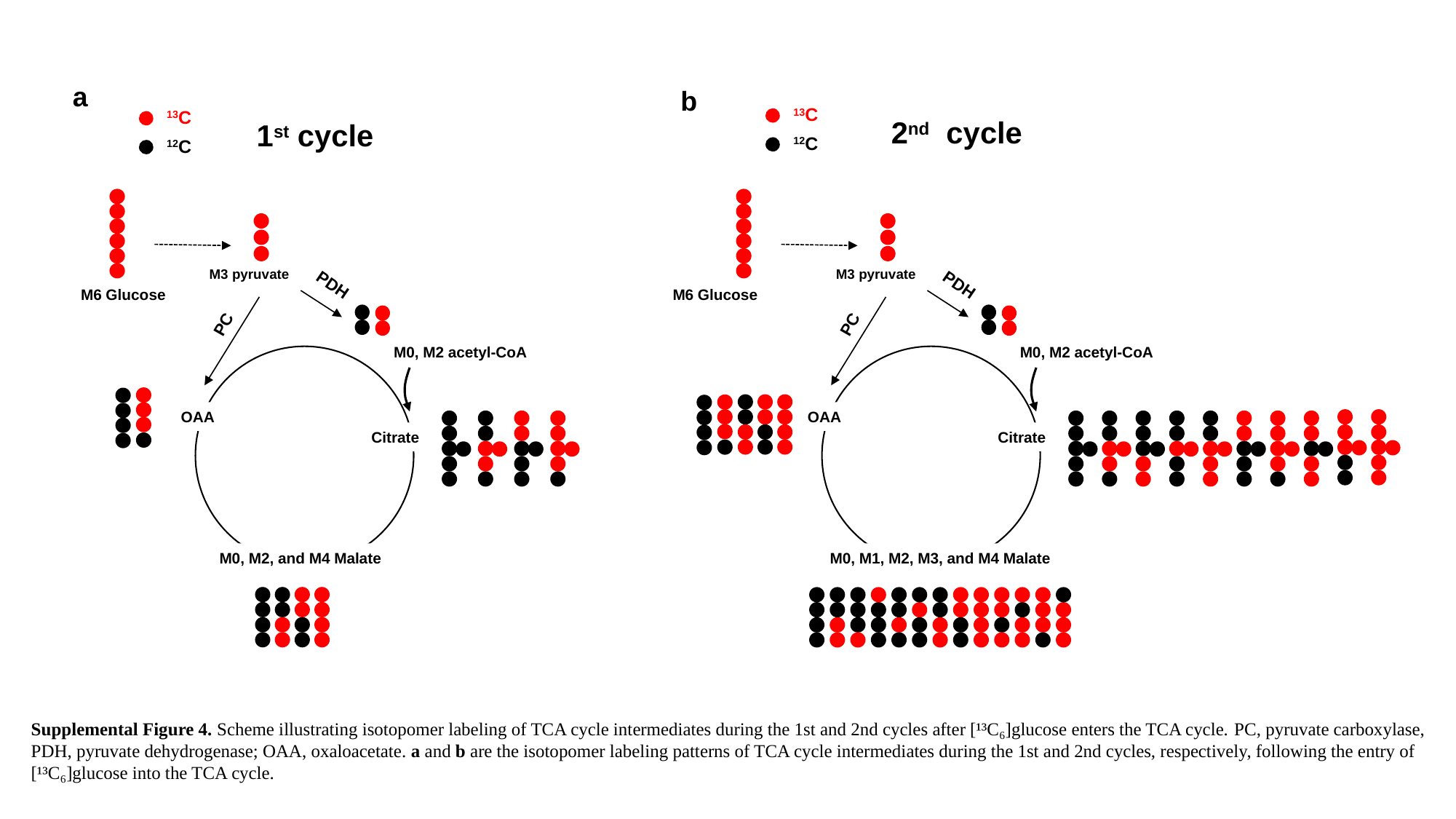

a
b
13C
13C
2nd cycle
1st cycle
12C
12C
M3 pyruvate
M3 pyruvate
PDH
PDH
M6 Glucose
M6 Glucose
PC
PC
M0, M2 acetyl-CoA
M0, M2 acetyl-CoA
OAA
OAA
Citrate
Citrate
M0, M2, and M4 Malate
M0, M1, M2, M3, and M4 Malate
Supplemental Figure 4. Scheme illustrating isotopomer labeling of TCA cycle intermediates during the 1st and 2nd cycles after [¹³C₆]glucose enters the TCA cycle. PC, pyruvate carboxylase, PDH, pyruvate dehydrogenase; OAA, oxaloacetate. a and b are the isotopomer labeling patterns of TCA cycle intermediates during the 1st and 2nd cycles, respectively, following the entry of [¹³C₆]glucose into the TCA cycle.

## Slide 5
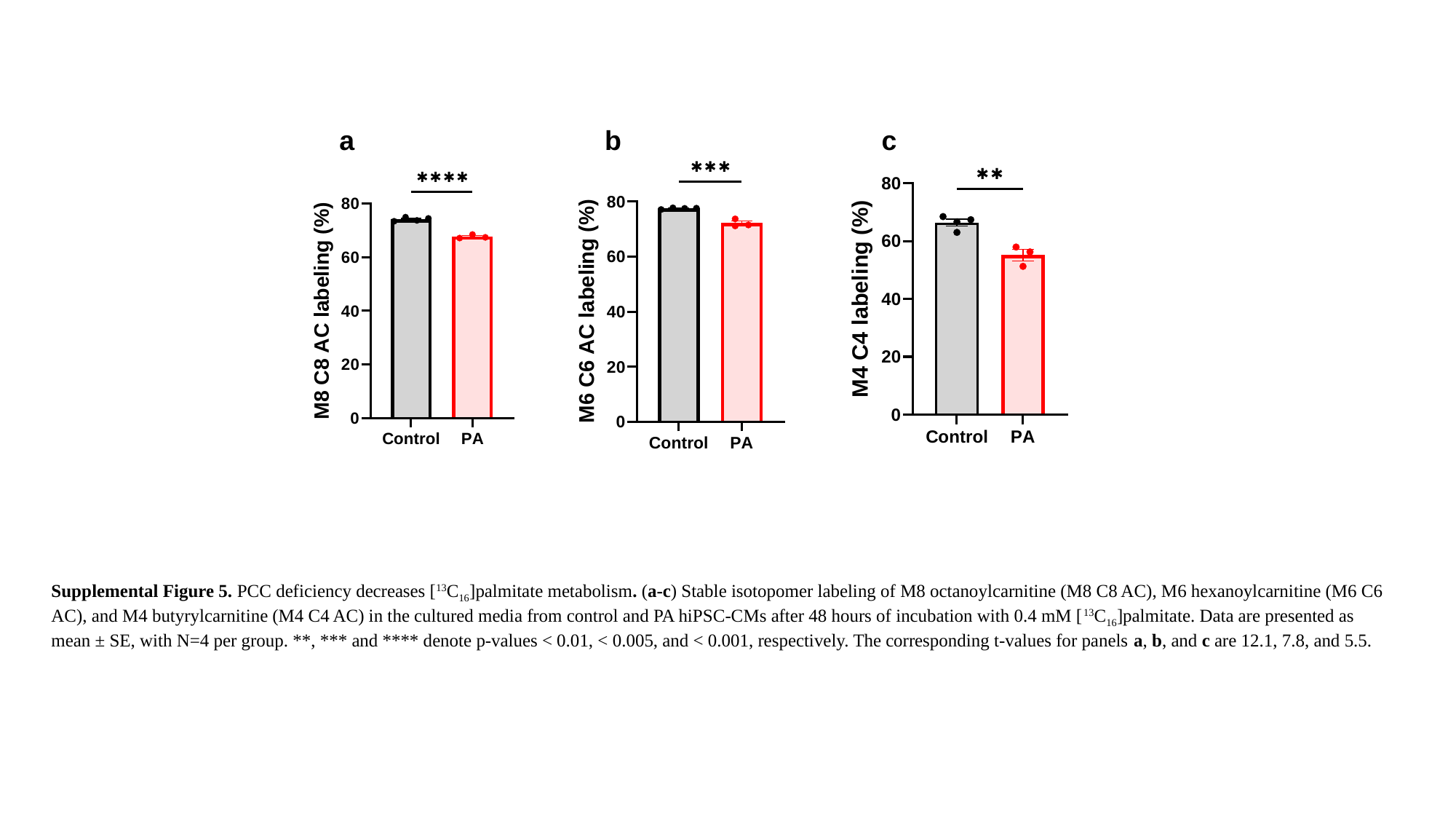

a
b
c
Supplemental Figure 5. PCC deficiency decreases [13C16]palmitate metabolism. (a-c) Stable isotopomer labeling of M8 octanoylcarnitine (M8 C8 AC), M6 hexanoylcarnitine (M6 C6 AC), and M4 butyrylcarnitine (M4 C4 AC) in the cultured media from control and PA hiPSC-CMs after 48 hours of incubation with 0.4 mM [13C16]palmitate. Data are presented as mean ± SE, with N=4 per group. **, *** and **** denote p-values < 0.01, < 0.005, and < 0.001, respectively. The corresponding t-values for panels a, b, and c are 12.1, 7.8, and 5.5.

## Slide 6
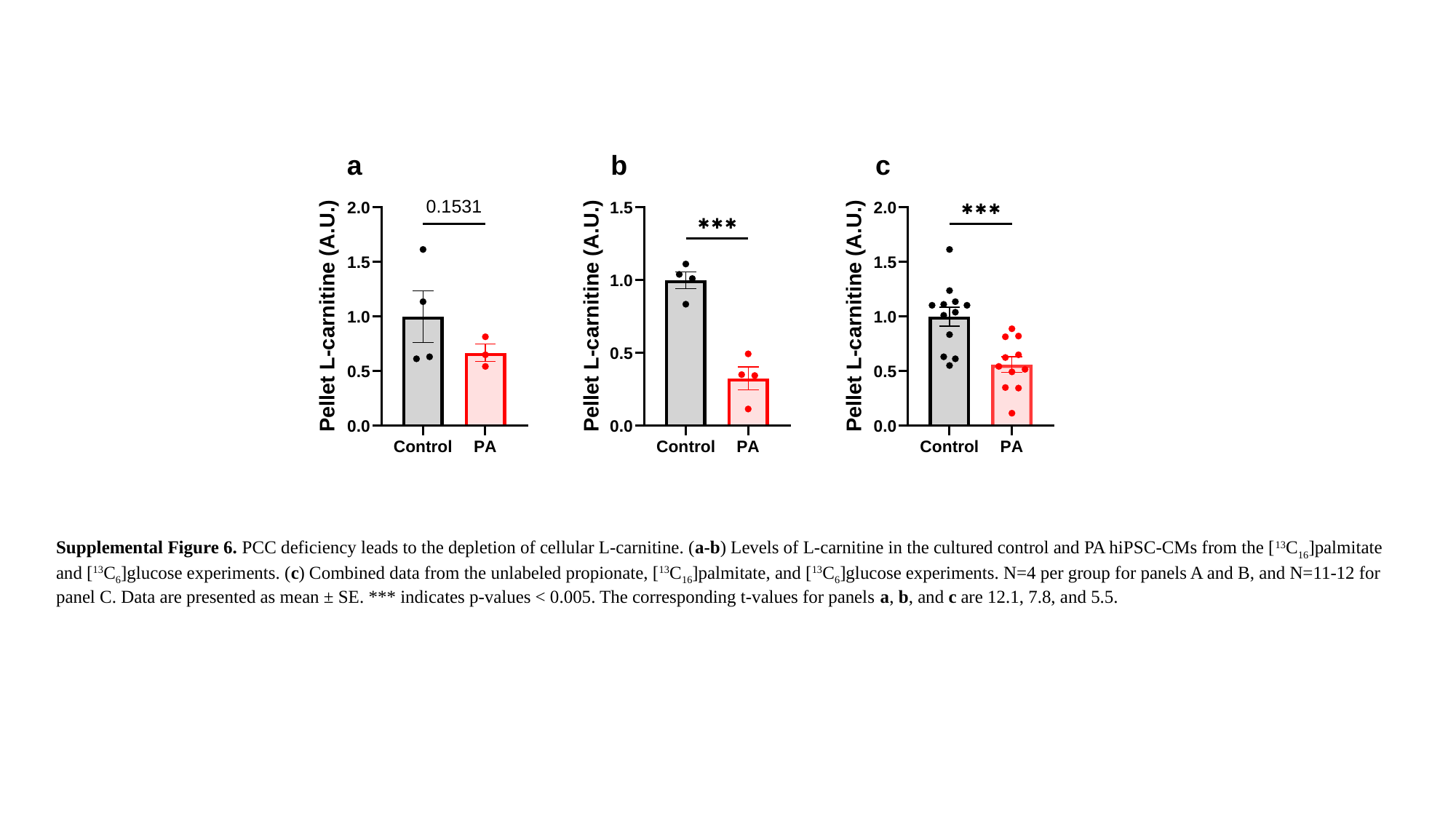

a
b
c
Supplemental Figure 6. PCC deficiency leads to the depletion of cellular L-carnitine. (a-b) Levels of L-carnitine in the cultured control and PA hiPSC-CMs from the [13C16]palmitate and [13C6]glucose experiments. (c) Combined data from the unlabeled propionate, [13C16]palmitate, and [13C6]glucose experiments. N=4 per group for panels A and B, and N=11-12 for panel C. Data are presented as mean ± SE. *** indicates p-values < 0.005. The corresponding t-values for panels a, b, and c are 12.1, 7.8, and 5.5.

## Slide 7
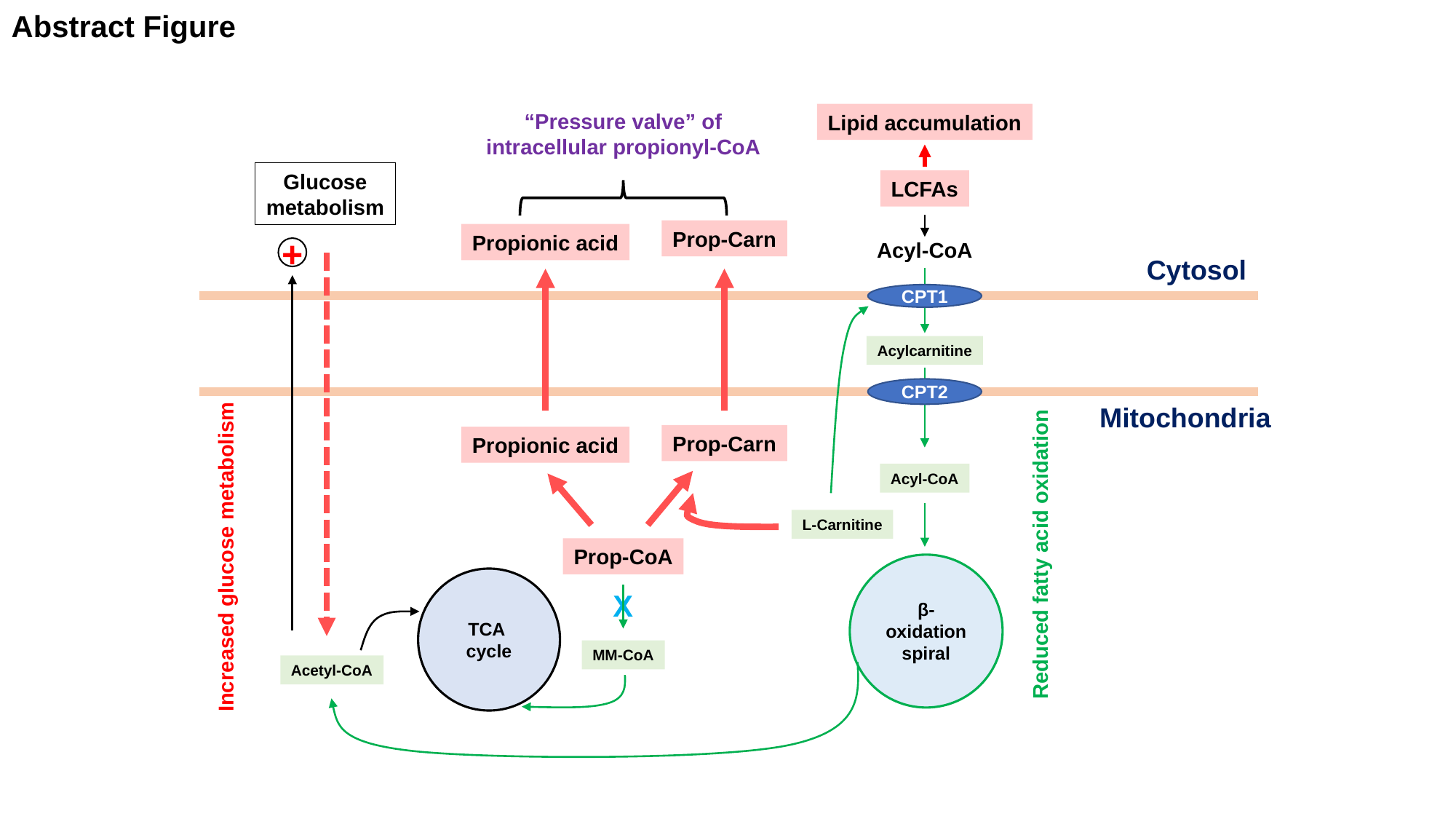

Abstract Figure
“Pressure valve” of
intracellular propionyl-CoA
Lipid accumulation
Glucose
metabolism
LCFAs
Prop-Carn
Propionic acid
+
Acyl-CoA
Cytosol
CPT1
Acylcarnitine
CPT2
Mitochondria
Prop-Carn
Propionic acid
Acyl-CoA
L-Carnitine
Reduced fatty acid oxidation
Prop-CoA
Increased glucose metabolism
β-oxidation
spiral
TCA
cycle
X
MM-CoA
Acetyl-CoA
